# Acquisition of chemical recognition cues facilitates integration into ant societies

**DOI:** 10.1186/1472-6785-11-30

**Published:** 2011-12-01

**Authors:** Christoph von Beeren, Stefan Schulz, Rosli Hashim, Volker Witte

**Affiliations:** 1 Department of Biology II, Ludwig Maximilian University Munich, Großhaderner Straße 2, Planegg-Martinsried, 82152, Germany; 2 Department of Organic Chemistry, Technical University Braunschweig, Hagenring 30, Braunschweig, 38106, Germany; 3 Institute of Biological Science, University Malaya, Kuala Lumpur, 50603, Malaysia

## Abstract

**Background:**

Social insects maintain the integrity of their societies by discriminating between colony members and foreigners through cuticular hydrocarbon (CHC) signatures. Nevertheless, parasites frequently get access to social resources, for example through mimicry of host CHCs among other mechanisms. The origin of mimetic compounds, however, remains unknown in the majority of studies (biosynthesis vs. acquisition). Additionally, direct evidence is scarce that chemical mimicry is indeed beneficial to the parasites (e.g., by improving social acceptance).

**Results:**

In the present study we demonstrated that the kleptoparasitic silverfish *Malayatelura ponerophila *most likely acquires CHCs directly from its host ant *Leptogenys distinguenda *by evaluating the transfer of a stable-isotope label from the cuticle of workers to the silverfish. In a second experiment, we prevented CHC pilfering by separating silverfish from their host for six or nine days. Chemical host resemblance as well as aggressive rejection behaviour by host ants was then quantified for unmanipulated and previously separated individuals. Separated individuals showed reduced chemical host resemblance and they received significantly more aggressive rejection behaviour than unmanipulated individuals.

**Conclusion:**

Our study clarifies the mechanism of chemical mimicry in a social insect parasite in great detail. It shows empirically for the first time that social insect parasites are able to acquire CHCs from their host. Furthermore, it demonstrates that the accuracy of chemical mimicry can be crucial for social insect parasites by enhancing social acceptance and, thus, allowing successful exploitation. We discuss the results in the light of coevolutionary arms races between parasites and hosts.

## Background

Host-parasite interactions are often regarded as coevolutionary "arms races" in which a host and a parasite species exert reciprocal selection pressures on one another over long periods of time [1]. Under coevolution, parasite species adapt towards encountering a host and exploiting it successfully, whereas host species in turn adapt towards an avoidance of parasite encounters or a successful defence against them [2]. Accordingly, hosts have evolved a great variety of defence mechanisms to prevent all sorts of exploitative attacks [3,4].

Since social insects are widespread and extraordinarily abundant [5] they are subject to exploitation. As a consequence, they have evolved sophisticated recognition systems to protect their colonies, their brood, and their resources from competitors, predators and parasites, thereby maintaining the integrity of their societies [6]. The recognition of group members in social insects is mainly based on chemical cues [7-9]. Individuals compare the chemical cues expressed by a counterpart with an internal template, which is the chemical signature expected in all members of the society [10]. Complex blends of cuticular hydrocarbons (CHCs) seem to comprise all essential information necessary for nestmate recognition in ants, wasps and termites [11]. Due to effective recognition systems, invaders are frequently recognized, attacked, expelled or even killed by social insect workers.

Nevertheless, a multitude of organisms, particularly invertebrates, are known to exploit social insect societies [12-14], for example, by preying directly on the host, by stealing their food, or merely by inhabiting a well-protected habitat with a stable microclimate [6]. Many of these organisms, commonly known as myrmecophiles, are more or less permanently associated with ant colonies [15]. However, tight associations with ants require specific adaptations, that is, intruders must be able to invade host colonies and maintain contact without being expelled or killed. Some species not only manage to invade ant societies successfully, they also remain permanently integrated [6,16,17] in a way that the hosts behave amicably to the intruders as if they were part of their society [18].

A range of specific strategies exist to penetrate ant societies, and eventually to remain permanently integrated, including chemical, acoustic, morphological and behavioural adaptations [6,10,19-22]. Chemical strategies are particularly widespread among myrmecophiles, most likely because ants rely strongly on chemical communication [5,23]. Several chemical strategies have been described, such as chemical mimicry (chemical resemblance of another species), chemical camouflage/crypsis (avoiding detection through expression of uninteresting or background cues), chemical insignificance (suppression of chemical recognition cues) or the use of ant deterrent/attractant chemicals [10,19]. Pretending to be a member of the colony by mimicking the ants' CHCs (chemical mimicry) is among the most frequent chemical strategies among myrmecophiles [10,23]. Although another definition of chemical mimicry exists [24], we use this term consistently with its original biological definition according to Dettner & Liepert [19], irrespective of the mechanism through which these mimetic compounds are acquired. Nevertheless, to include this information, we consider chemical mimicry to be either innate (biosynthesis of compounds), acquired (adoption of compounds), or mixed.

**Figure 1:**
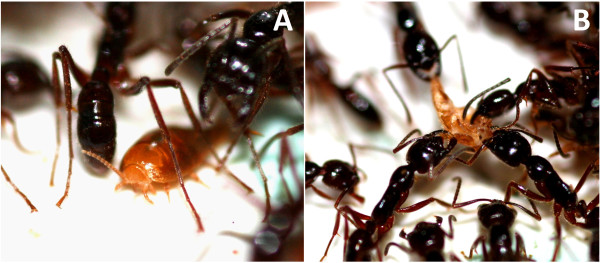
**Interactions of *M. ponerophila *with host workers**. (A) The silverfish is frequently found beneath host ant workers. Physical contact with the host ants allows the silverfish to acquire cuticular hydrocarbons, which are used by ants as recognition cues. (B) Life in ant colonies also entails a high risk for silverfish, as they are sometimes recognised, attacked, and killed by the host ants. ^© ^C. von Beeren.

Regarding the origins of mimetic compounds, we assume that the acquisition as well as the innate production of ant CHCs may be associated with costs for the myrmecophiles. As expected from a trade-off model, such costs must be balanced by a benefit of performing chemical mimicry [25], and this has rarely been tested empirically. In numerous cases, chemical mimicry presumably works through acquisition of host odours through physical contact rather than through biosynthesis [19,23]. However, the origin of mimetic compounds remains unclear in the majority of cases. As in many other examples, the kleptoparasitic silverfish *Malayatelura ponerophila *(Zygentoma, Atelurinae; Figure 1) was found to resemble the CHC profiles of its Southeast Asian army ant host, *Leptogenys distinguenda*. A closer analysis suggested the acquisition of host cues through physical contact, as the silverfish was observed to interact frequently with its host through rubbing its surface on that of host ant workers [26]. Nevertheless, final proof was lacking so that the mechanism remained speculative and a biosynthesis of mimetic cues could not be completely ruled out, as is the case in most other examples.

The aims of the present study were twofold; 1) to clarify the underlying mechanism of chemical mimicry (innate vs. acquired), and 2) to test whether a good match of chemical host recognition cues is in fact beneficial to the mimic (e.g., by facilitating social integration). Two experimental approaches were used to address these questions. First, we marked host ant workers with a stable isotope-labelled hydrocarbon and monitored the transfer of this artificial label to the myrmecophilous silverfish. If *M. ponerophila *acquires CHCs from its host, we expected that the label would also accumulate on their cuticles. Second, we aimed to experimentally reduce the chemical host resemblance of silverfish individuals in order to study the effects on social integration. Therefore, silverfish were isolated from their host ants for six and nine days, respectively. Under the assumption of a behavioural acquisition of host recognition cues by the parasite, the silverfish were expected to lose host CHCs over time when isolated, resulting in reduced chemical similarity to the host. This assumption was checked by analysing cuticular chemical profiles of isolated and non-isolated silverfish and comparing them to their host. We expected that silverfish exhibiting reduced chemical host resemblance would be less socially integrated as a consequence of being increasingly recognized as alien. Hence, we tested through behavioural studies whether isolated silverfish (with reduced host similarity) were attacked more frequently than unmanipulated individuals.

## Methods

### (a) Field collection and animal maintenance

Animals were collected and observed at the Field Studies Centre of the University Malaya in Ulu Gombak, Malaysia (03°19.479' N, 101°45.163' E, altitude 230 m) and at the Institute of Biodiversity in Bukit Rengit, Malaysia (03°35.779' N, 102°10.814' E, altitude 72 m). Ten months of field work were carried out in total between August 2008 and April 2011. We searched for *L. distinguenda *raiding trails during the night, tracked them back to their bivouac-like nests and subsequently checked every 30 min between 8 p.m. and 3 a.m. for the onset of a migration. The silverfish *Malayatelura ponerophila *[27] participate in migrations either by phoretic transport on host pupae or by following the ants' pheromone trail on their own [28]. The collected animals were either extracted directly for subsequent chemical analysis of CHCs, or they were maintained in artificial laboratory nests for various experiments (see below). Experimental colonies were assembled differently depending on the numbers of collected individuals and on the experimental protocol. Laboratory nests contained only members from one particular host colony (i.e., colonies were never mixed). If not described differently, nests consisted of a clear plastic container (20 × 14 × 1 cm), shaded with a plastic cover, and with a 1.5 cm wide entrance. The nest was placed in a larger foraging arena (32 × 25 × 9 cm) with a moistened plaster floor to maintain constant humidity. For isolation experiments animals were also kept separated from their home colonies in plastic containers (20 × 14 × 5 cm) equipped with a moist plaster floor. Animals in laboratory nests and those in isolation were fed every day with freshly killed crickets. Crickets are among the natural diet of the host ants [29] and the silverfish (personal observation). All behavioural studies were performed between 8:00 p.m. and 3:00 a.m. under dim scattered light since the focal animals are strictly nocturnal.

### (b) Chemical transfer experiment

The chemical transfer experiment was carried out to test whether silverfish acquire CHCs from their host through physical contact. One hundred mature workers (collected from raids), 60 callows (newly hatched workers), approximately 40 larvae, 30 pupae, 21 silverfish and 10 non-myrmecophilous isopods (as a control) were kept in a nest constructed from natural materials (soil and leaf litter). The callows were treated with a stable isotope-labelled hydrocarbon (eicosane-d_42_, C/D/N Isotopes Inc., Canada, Pointe-Claire). Callows were selected for the labelling treatment because they are less mobile [30] and less aggressive (CvB and VW, personal observation), and silverfish were found to interact preferentially with them [26]. Eicosane-d_42 _was used as a label because it has properties (chain length, molecular weight) similar to the CHCs that occur naturally on the host ants [26]. 200 μl of a saturated eicosane-d_42 _hexane solution were evaporated in a clean 20 ml glass vial so that the hydrocarbon fully covered the bottom of the vial as a solid film. The callows were then enclosed in the vial, which was moved gently for 30 min to transfer the labelled compound. Callows did not visibly suffer from this treatment. Labelled callows, untreated workers, silverfish, and control isopods were kept three days together in the laboratory nest and subsequently they were extracted for chemical analysis (details below). The isopods, collected from the natural habitat, were added to test whether eicosane-d_42 _transmits to animals in the nest environment that are not specifically in close contact with the host. Preliminary studies revealed that isopods were ignored by ants, which makes them ideal control animals. Ten additional isopods were directly labelled with eicosane-d_42 _as described above (labelled isopods) and extracted to verify that the isopod cuticle is able to adsorb the labelled compound.

### (c) Isolation treatments

To manipulate the presence of host CHC profiles on silverfish (under the assumption of acquisition through physical contact) and to test for effects on behavioural interactions with the host ants, silverfish and host ants (as experimental and control groups, respectively) were separated from their home colonies and kept isolated for six (6d) or nine days (9d) (for sample sizes refer to table 1). Isolated and non-isolated individuals as well as some resident host workers were either extracted with hexane to analyse changes in their CHCs (colonies 1-3; see section (d) of the methods) or they were tested behaviourally in their home colonies for social acceptance (colonies 4-6; see section (e) of methods), or both in combination (colony 7; see section (f) of the methods). The combined experiment (section (f) of the methods) was best suited for testing whether an individual's accuracy of chemical mimicry affects its level of social acceptance. The chemical (section (d) of the methods) and behavioural effects (section (e) of methods) were in addition studied independently, as an influence on the parasites' CHC signature through host contact during behavioural tests cannot be ruled out in the combined experiment.

**Table 1:** Overview of the number of silverfish individuals observed within each colony for the analysis of CHCs, the social acceptance experiment and the measurement of the silverfish' body surface area.

Colony	Analysis of CHCs		Social acceptance experiment		Body surface area	
	No isolation	Isolation	No Isolation	Isolation	No Isolation	Isolation
Colony 1	15	29**	-	-	15	27**
Colony 2	15	15**	-	-	15	15**
Colony 3	12	12*	-	-	10	12*
Colony 4	-	-	7	6*	-	-
Colony 5	-	-	18	24*	-	-
Colony 6	-	-	6	8*	-	-
**Accuracy of chemical mimicry**						
Colony 7	21	14**	22	15**	21	14**

The number of silverfish individuals differs in some colonies between the multivariate analysis of all behavioural categories and the analysis of the aggression index as some individuals did not complete the standardised number of 50 ant contacts for the social acceptance experiment as some silverfish were seized by ant workers (Table 2). Abbreviations: * = six days isolated silverfish; ** = nine days isolated silverfish

A control experiment was performed with silverfish to determine whether the isolation treatment itself had an effect on their social acceptance, rather than changes in their CHC signature (e.g., due to physical suffering or adoption of additional compounds that originate from the experimental setup). As before, silverfish were isolated (for six days), and then one group was directly subjected to aggression tests and subsequently to chemical analyses, while the other group was allowed an additional 24 h contact with 50 host callows, before they were tested in the same way. The latter group thereby experienced the isolation treatment but was also given the chance to re-acquire host CHCs (silverfish isolation control; colony 8). Additional isolation control experiments were performed with adult ant workers collected from raids. The ant worker controls were conducted with three different colonies (colonies 9-11). These controls intended to test whether an isolation treatment similarly affects the expression of host worker CHC profiles (given the fact that they are able to biosynthesise the CHCs).

### (d) Analysis of CHCs

Specimens were transferred individually into 2 ml vials with PTFE septa and extracted for 10 min in 200 μl hexane (HPLC grade, Sigma-Aldrich). After evaporation of the solvent, the CHCs were re-dissolved in 40 μl hexane containing an internal standard (methyl stearate, FLUKA Analytics, Sigma-Aldrich), and 20 μl were transferred into a 0.3 ml vial with limited volume insert (Chromacol, 03-FISV). Using an auto sampler (Agilent technologies, 7683 Series) 1 μl of each sample was injected into a gas chromatograph (Agilent Technologies 6890N) coupled to a mass spectrometer (Agilent Technologies GC 5975 MSD). Details on the methods can be found in [28].

Chemicals were identified by mass spectra and retention indices (RI), and peak areas were extracted using the software AMDIS (version 2.68) [31]. A target library of 109 compounds was created based on the compounds found on host ants and myrmecophiles [26]. As AMDIS uses the mass spectrum as well as the retention index to identify a substance, it has the advantage of reliably detecting compounds, even in low quantities. Structural alkene isomers were distinguished although double bond positions were not determined.

The absolute quantity of each compound was calculated using the internal standard (concentration = 20 ng/μl). The resulting total quantity of a sample was divided by the animals' surface area in square millimetres in order to standardise to a presumably perceivable concentration of chemicals by an ant's antennal contact and to control for size differences between animals. To calculate surface areas, the bodies of silverfish, workers and isopods were subdivided into geometrical areas and the relevant dimensions were measured using a stereomicroscope (Zeiss Stemi 2000-C) with a measuring eyepiece (see additional file 1: Calculation of animal surface areas). The surface area of silverfish was calculated for each individual separately because they varied considerably in size, while a median surface area was used for workers as well as for isopods. Specimens were stored in pure ethanol.

### (e) Social acceptance experiments

The host's aggression toward individual silverfish or individual workers was quantified through a standardised contact study in laboratory nests. The nests contained 200 ant workers, which were collected from raids, because foraging workers behave more aggressively and are thus more likely to defend the colony [32].

Furthermore, the ants were given 1 h time to settle in the laboratory nest before starting the experiments because ants tend to behave more aggressively in familiar territory than in an unfamiliar setting [33]. Fifty consecutive encounters of a silverfish individual (or worker individual) and ants were then categorized according to table 2. Each individual was tested only once. However, repeated interactions with the same ant individuals were possible. Nevertheless, since we focused on a colony-level defence, which naturally includes task allocation, repeated interactions of the same workers do not affect our interpretations. An aggression index (AI) was calculated for each silverfish from the observed interactions as follows: AI = N_A_/N_T _with N_A _= number of aggressive interactions and N_T _= total number of interactions.

**Table 2:** Behavioural interactions between silverfish and ants and behavioural categories used for calculating the aggression index.

Behaviour	Definition	Category
Ignored	An ant worker touches the silverfish once with its antennae and moves on without any sign of behavioural modification.	-
Groomed	An ant grooms the silverfish with its mouthparts. The silverfish remains in position.	-
Avoid	When an ant approaches, the silverfish avoids contact by quick escape.	-
Antennated	An ant touches a silverfish repeatedly with its antennae for longer than two seconds without displaying other behaviours.	-
Unnoticed	An ant comes into and perhaps stays in contact with a silverfish, but not with its antennae; the ant does not modify its behaviour.	-
Chased	An ant touches the silverfish with its antennae and quickly lunges in its direction.	Aggressive
Snapped	An ant touches the silverfish with its antennae and snaps with its mandibles in the direction of the silverfish.	Aggressive
Stung	An ant touches the silverfish with its antennae, lunges forward and bends its gaster in the direction of the opponent. The attempt is not necessarily successful.	Aggressive
Seized	An ant snapped at and subsequently seized a silverfish in its mandibles.	Aggressive

Some silverfish (*N *= 14) were seized by the ants before 50 encounters were completed. These individuals were removed to prevent their destruction so that they could be used for chemical analysis and body measurements. Although these individuals did not reach 50 host encounters, their AI was calculated as described above.

### (f) Accuracy of chemical mimicry

To directly test the relation of chemical host resemblance to social acceptance for the same individuals, we combined the social acceptance study and the analysis of CHCs in one *L. distinguenda *colony (colony 7; table 1). For each silverfish individual host aggression was quantified first via social acceptance experiments (standardised contact study), and then its CHCs were extracted and subjected to chemical analysis.

### (g) Data analysis

Chemical and behavioural data were evaluated with the software PRIMER 6 (version 6.1.12, Primer-E Ltd., Ivybridge, U.K.) with the PERMANOVA+ add-in (version 1.0.2) using a non-parametric permutational analysis of variance (PERMANOVA) with 9,999 permutations [34]. PERMANOVA models were based on Bray-Curtis similarities (as a semi-metric measure), either calculated from a single response variable (chemical similarity, CHC concentration, aggression index), or from numerous response variables (CHC profiles, behavioural interactions). Nonmetric multidimensional scaling (NMDS) was used to visualise multivariate data (PRIMER 6). Box plots were created from univariate data with the Microsoft Excel add-in SSC-Stat (version 2.18, Statistical service centre of the University of Reading, Reading, U.K.). Chi-square tests were accomplished using XLSTAT (Version 2010.3.06, Addinsoft, U.S.A.).

#### Chemical analysis

Since no silverfish-specific compounds were detected, the principle compounds that together constituted 99% to the chemical profiles of workers (*N *= 44) according to a similarity percentage analysis (SIMPER) on Bray-Curtis similarities were included in the statistical analysis of the CHC composition, the presence or absence of CHCs, the total CHC concentration and the chemical similarity (*N *= 32 compounds; see additional file 2: Table of compounds). To test whether the chemical similarity of silverfish to their host colony was influenced by isolation treatments, Bray-Curtis similarities to the average worker CHC profile of the respective host colony were used as a univariate response variable, and a PERMANOVA with a 2-factor nested design (colonies (random), days of isolation (fixed), nested in colony) was applied. No chemical worker profiles were available for colony 3. To test for additional differences in the quantity of CHCs, absolute concentrations (per surface area) were analysed in the same way.

Furthermore, multivariate approaches were used to analyse relative changes in CHC composition (Bray-Curtis similarities), and the presence or absence of compounds (simple matching). A PERMANOVA with a 2-factor nested design as described above was applied for both analyses. Chromatograms of chemical profiles of host ants and silverfish can be found in an earlier article of one of the authors [26].

#### Behavioural analysis

Aggression indices of isolated vs. non-isolated individuals were compared using a PERMANOVA with a two-factor nested design as described above. The interactions of silverfish with their host ants were evaluated in a multivariate approach including all observed behaviours. These were standardised by the total number of interactions and a 2-factor nested design as described above was applied.

## Results

### (a) Chemical transfer experiment

**Figure 2:**
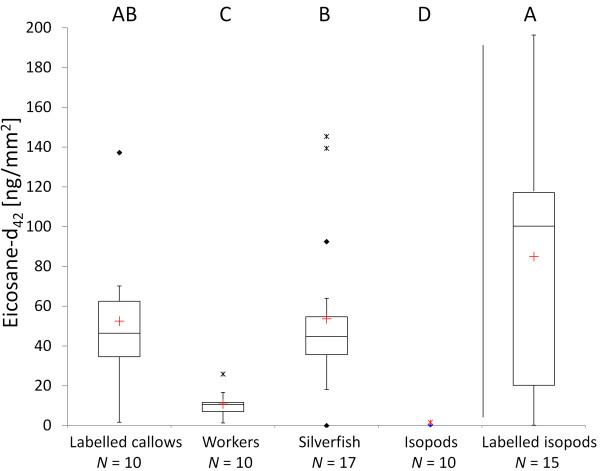
**Concentrations of eicosane-d_42 _in the CHC transfer experiment**. Different capital letters show significant differences (*P *< 0.05) between groups evaluated by PERMANOVA. Median (red cross = mean), quartiles (boxes), 10^th ^and 90^th ^percentiles (whiskers), and outliers (black square = outlier, asterisk = extreme point) are shown.

Previously labelled callows still carried high concentrations of eicosane-d_42 _after the three-day experimental phase (median = 46.18 ng/mm^2^; Figure 2). Interestingly, the concentration of eicosane-d_42 _did not differ between silverfish (median = 44.57 ng/mm^2^) and callows (median = 46.18 ng/mm^2^; PERMANOVA, *P *= 0.986), while lower concentrations were found on adult workers (median = 10.60 ng/mm^2^; PERMANOVA, for both comparisons *P *< 0.001). Almost no eicosane-d_42 _was found on control isopods (median = 0 ng/mm^2^), which consequently differed from labelled callows, workers and silverfish (PERMANOVA, for all comparisons *P *< 0.001). High quantities of eicosane-d_42 _on the labelled isopods (median = 100.13 ng/mm^2^) demonstrated that their cuticle has the potential to adsorb the labelled CHC.

### (b) Analysis of CHCs

Seventy compounds were detected on workers (*N *= 44). No silverfish-specific CHC was found (N = 133). The number of detected host compounds on silverfish decreased after isolation treatments (no isolation: *N*_compounds _= 28, *N*_silverfish _= 63; 6 days isolation: *N*_compounds _= 22, *N*_silverfish _= 12; 9 days isolation: *N*_compounds _= 23, *N*_silverfish _= 58). No compounds were detected on some of the specimens that had been isolated for 9 days (5 out of 58).

**Figure 3:**
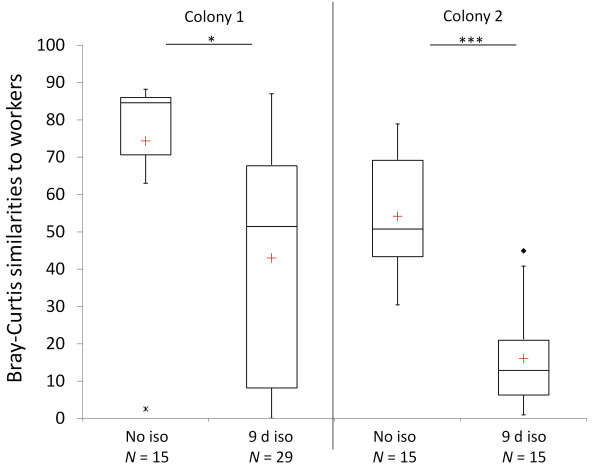
**Chemical similarities of individual silverfish to the average chemical worker profile of their host colony (*N*_workers _≥ 10)**. No chemical worker profiles were available for colony 3. Differences between isolated vs. non-isolated silverfish were evaluated by PERMANOVA (*P < 0.05, ***P < 0.001). Median (red cross = mean), quartiles (boxes) 10^th ^and 90^th ^percentiles (whiskers), and outliers (black square = outlier; asterisk = extreme point) are shown. Abbreviations: No iso = no isolation, d iso = days isolated.

Non-isolated silverfish were chemically closer to their host workers than isolated individuals (PERMANOVA, for all colonies *P *≤ 0.025; Figure 3). Significant differences between isolated and non-isolated silverfish were detected in the relative composition, the presence or absence, and in the total concentration of CHCs in three out of four different colonies (table 3). In colony 3 there was a trend that the compositions of CHCs differed between non-isolated and isolated silverfish (PERMANOVA, *P *= 0.064), whereas the presence or absence of CHCs and the CHC concentrations did not differ (PERMANOVA, *P *≥ 0.134). For this colony we had the smallest sample size (see table 1).

**Table 3:** Comparison of non-isolated silverfish (0 d) and isolated silverfish (6 d or 9 d) regarding their CHC composition, presence or absence of CHCs and their total CHC concentration.

Colony	CHC	CHC	CHC
(Isolation of silverfish)	composition	presence/absence	concentration
Colony 1 (0 d vs. 9 d)	0.015	0.001	0.001
Colony 2 (0 d vs. 9 d)	0.010	0.001	0.001
Colony 3 (0 d vs. 6 d)	0.064	0.801	0.134
Colony 7 (0 d vs. 9 d)	0.001	0.002	0.005

PERMANOVA *P *values are shown. For sample sizes see table 1. Abbreviations: d = days isolated

Non-isolated silverfish carried higher total concentrations of host CHCs on their body (median = 55.23 ng/mm^2^, *N *= 40) than isolated silverfish (median _6 days _= 10.95 ng/mm^2^, *N *= 12; median _9 days _= 13.98 ng/mm^2^, *N *= 42; PERMANOVA, *P *< 0.001; for within colony comparisons see additional file 3: Concentrations of CHCs). Workers carried significantly higher concentrations than both silverfish groups (median = 106.23 ng/mm^2^, *N *= 44; PERMANOVA, *P *< 0.001). Across all colonies the median concentration of every compound was lower after isolation.

### (c) Social acceptance experiment

**Figure 4:**
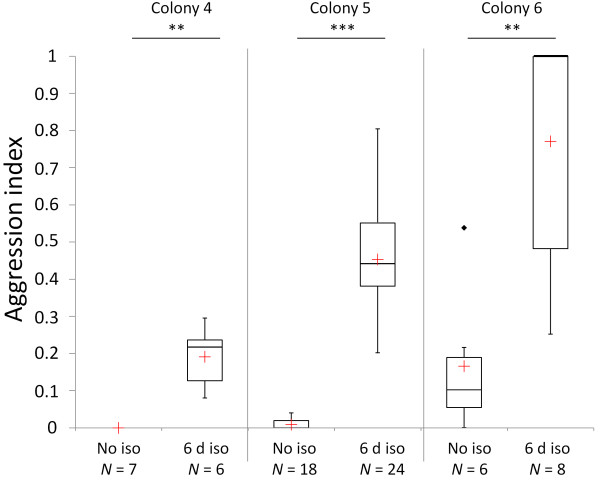
**Host aggression in three different colonies toward non-isolated silverfish and silverfish that were isolated for six days**. Differences between groups were evaluated by PERMANOVA (**P < 0.01, ***P < 0.001). Median (red cross = mean), quartiles (boxes), 10^th ^and 90^th ^percentiles (whiskers), and outliers (black square = outlier) are shown. Abbreviations: No iso = no isolation, d iso = days isolated.

In all colonies, isolated silverfish were treated with higher aggression by host workers than non-isolated silverfish (PERMANOVA, for all comparisons *P *≤ 0.004; Figure 4). The higher aggression toward isolated silverfish was also reflected in the frequency with which they were seized by workers. Only 4% of non-isolated silverfish were seized, while 26% of the six day isolated and 20% of nine-day isolated individuals were seized. All isolated silverfish were seized by workers in colony 6. The frequencies of seized and non-seized silverfish did not differ between six and nine days isolated silverfish (Chi square test: *χ^2 ^*= 0.232, *df *= 1, *P *= 0.630, *N_1 _*= 38, *N_2 _*= 15), but they differed significantly between non-isolated and isolated individuals (Chi square test: *χ^2 ^*= 11.851, *df *= 1, *P *= 0.001, *N_1 _*= 53, *N_2 _*= 53).

Considering the multivariate analysis of all behavioural interactions, we found significant differences in three of four colonies (colony 4, 5 and 7) between non-isolated and isolated silverfish (PERMANOVA, for all pair-wise comparisons *P *≤ 0.012). Colony 6 was not evaluated because all of the isolated individuals were seized by worker ants and could not complete the standardised number of 50 ant contacts. For detailed information on behavioural interactions across all colonies see additional file 4: Behavioral interactions.

### (d) Accuracy of chemical mimicry

**Figure 5:**
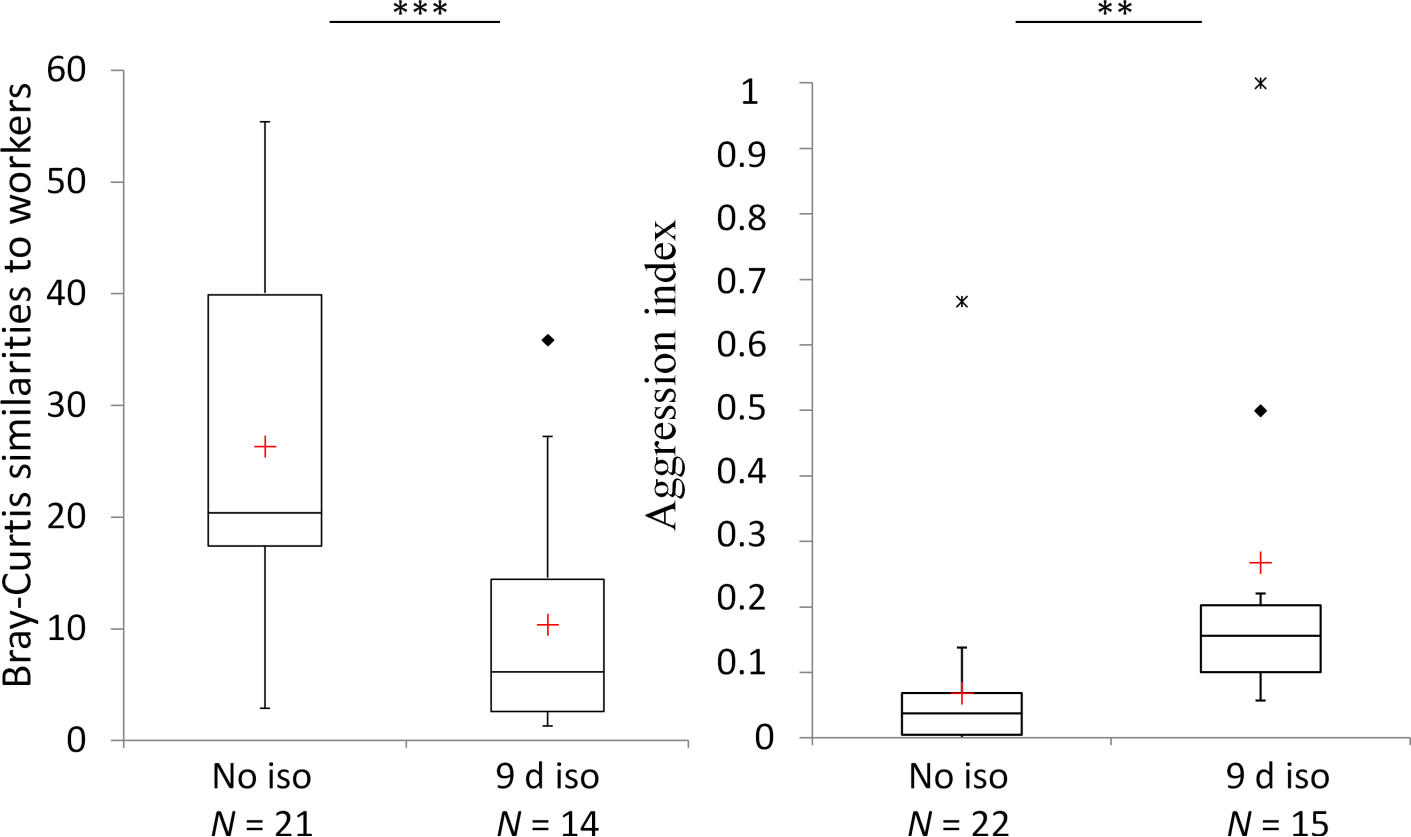
**Chemical similarities of silverfish to the average chemical worker profile (N_workers _= 19; left) and host aggression toward the same individuals (right) in colony 7**. Differences between isolated and non-isolated silverfish were evaluated by PERMANOVA (**P < 0.01, ***P < 0.001). Median (red cross = mean), quartiles (boxes), 10^th ^and 90^th ^percentiles (whiskers), and outliers (black square = outlier; asterisk = extreme point) are shown. Abbreviations: No iso = no isolation, d iso = days isolated.

In the experiment on the accuracy of chemical mimicry, the cuticular profile of isolated silverfish was also less similar to host workers (PERMANOVA, *P *< 0.001) and the same silverfish individuals received more aggression in contact studies than non-isolated individuals did (PERMANOVA, *P *≤ 0.004; Figure 5). As in the experiments described above the total concentration of CHCs was lower in isolated silverfish (median = 4.51 ng/mm^2^, N = 21; PERMANOVA, *P *< 0.001) than in non-isolated silverfish (median = 27.66 ng/mm^2^, N = 21). Furthermore, non-isolated silverfish remained unnoticed more often and were ignored more frequently compared to isolated individuals (see additional files 4 and 5). Isolated silverfish were more frequently antennated by host workers, and they avoided host contact more often than non-isolated silverfish. Most importantly, ant workers chased and snapped at isolated silverfish more frequently than at non-isolated silverfish. There were no significant differences in the interactions "groomed" (PERMANOVA, *P *= 0.364) and "stung" (PERMANOVA, *P *= 0.365) between isolated and non-isolated silverfish (see additional file 4).

In the isolation control experiment silverfish that were first isolated for six days and then kept together with callows for 24 h showed greater chemical similarity (PERMANOVA, *P *< 0.001) and were treated less aggressively (PERMANOVA, *P *< 0.001) than individuals that were only isolated but had no secondary contact to the host (see additional file 6: Silverfish isolation control experiment). In the host worker isolation control experiment, CHC concentration did not decrease after isolation, instead it increased in two colonies (additional file 7: Worker control experiment). Furthermore, aggressive behaviour did not increase (no isolation: three aggressive interactions from a total of 2282 recorded interactions; nine days isolation: five aggressive interactions from a total of 2777 recorded interactions).

## Discussion

The present study sheds light on two important aspects of social insect parasitism. Our results strongly indicate that mimetic CHCs are acquired by a parasite from the cuticle of its host and that higher accuracy in mimicking host CHCs can be crucial for social exploitation due to the avoidance of aggressive rejection. In the following paragraphs we discuss in detail the integration mechanism of the parasitic silverfish *M. ponerophila*.

### Origin of mimetic compounds (acquisition vs. biosynthesis)

The adoption of a stable isotope-labelled hydrocarbon from the cuticle of the host by the silverfish but not by control animals indicates that silverfish use a behavioural mechanism for acquiring mimetic CHCs, rather than innate biosynthesis. Eicosane-d_42 _has properties (chain length, molecular weight) similar to CHCs that occur naturally on the host ants, thus we conclude that the hosts' natural surface compounds are acquired by the same mechanism. Although we did not directly tested natural host CHCs, these compounds are most likely transferred in the same way. We cannot imagine a mechanism by which silverfish acquire selectively only particular compounds from the host cuticle. Furthermore, the mechanism of pilfering host CHCs (e.g., rubbing behaviour [26]; see also additional file 8: Video of *M. ponerophila*) appears to be very effective, as silverfish accumulated even higher concentrations of eicosane-d_42 _from the labelled callows than host workers did. In agreement with a behavioural adoption, the mimetic CHCs on silverfish decreased in isolation treatments quantitatively (total concentration) and qualitatively (relative abundance and presence or absence). Importantly, mimetic CHCs increased again after secondary contact of previously isolated silverfish with host ants. Taken together, the loss of mimetic cues after isolation and their re-occurrence after secondary host contact point strongly towards an effective behavioural acquisition of host CHCs. Alternatively, these findings could be explained by a context-specific up- and down-regulation of CHC biosynthesis in the silverfish. However, due to the direct transfer of the labelled compound from host to silverfish, and due to evolutionary considerations that we explain below, this seems highly unlikely.

The exchange of surface compounds through physical contact (trophallaxis, allogrooming and/or other contact) has previously been demonstrated to occur among ant nestmates [35] but not between host ants and their myrmecophiles. Even though previous studies have been founded on the assumption that myrmecophiles acquire rather than synthesise mimetic compounds to achieve chemical resemblance [36-38], acquisition has never been clearly demonstrated. A loss of host-specific surface compounds after isolation has already been demonstrated in the beetle *Myrmecaphodius excavaticollis *[36] as well as in the cricket *Myrmecophilus *sp. [39]. These results render the biosynthesis of host CHCs in these myrmecophiles unlikely, but a potential ability to down-regulate the biosynthesis of host-specific CHCs in the absence of a host cannot be ruled out. Such ability was found in the myrmecophilous butterfly *Phengaris *(*Maculinea*) *rebeli *[40].

*Phengaris *(*Maculinea*) *rebeli *caterpillars biosynthesise a subset of their host's hydrocarbons to become attractive, resulting in the transport into the nests [41]. Importantly, for this mechanism to work, the allomones produced by innate biosynthesis must be colony-unspecific. Indeed, *Phengaris *(*Maculinea*) caterpillars seem to mimic the surface chemistry of ant brood [41,42], which is generally less complex compared to that of workers and is assumed to be colony-unspecific. Hence, appropriate cues may mimic, for example, certain key stimuli of brood or males [38,40]. We presume that the more complex a host's recognition signature is, the more difficult it becomes for distantly related organisms to evolve the appropriate biosynthetic pathways for the production of the essential recognition cues and to express the compounds in the correct relative proportions (even if key regulatory enzymes are involved). In such cases, mixed strategies or the adoption of recognition cues may be evolutionarily more parsimonious. Another problem associated with the biosynthesis of mimetic cues is the dynamic nature of colony specific CHC profiles. An ant species is typically characterized by a set of CHCs, which differ among colonies in relative proportions [9]. Hydrocarbons are exchanged between nestmates by means of trophallaxis (exchange of nutritional liquids between nestmates) and allogrooming (grooming directed towards a nestmate), which establishes a uniform colony odour-the "gestalt odour" [9,43]. Despite its uniformity, the "gestalt odour" changes over time due to factors such as shifts in diet [44,45], different nesting materials [46] or seasonal differences [47]. Biosynthesis of worker CHCs is unlikely to be able to adjust to such flexible but specific "gestalt odours". These considerations may explain why an acquisition of mimetic CHCs is found more frequently than biosynthesis among distantly related parasites of social insects.

### The role of accuracy in chemical mimicry

In addition to the mechanism of acquired chemical mimicry, our results highlight the importance of accuracy in chemical host resemblance by demonstrating that aggressive rejection can be avoided through closer chemical resemblance to the host. Notably, a parasites' successful social integration by chemical mimicry needs to include in principle only the cues that are necessary for nestmate recognition and not all the host CHCs. Nestmate recognition in the ant species *Formica exsecta*, for example, seems to be based only on selected compounds [48]. All types of compounds present on the cuticles of the host ant *L. distinguenda *and its parasitic silverfish could potentially be involved in recognition. Due to the generally accepted role of CHCs in ant nestmate recognition, we focused on non-polar compounds by using an appropriate solvent. There were several host CHCs on the silverfish, but only traces of other compounds (see additional file 2). Since host aggressiveness apparently depended on the chemical similarity of silverfish to their host, we conclude that the chemical recognition of silverfish by the host is predominantly based on CHCs. However, we were not able to differentiate which characteristics of CHC profiles, i.e. the composition (relative proportions), the presence or absence or the concentrations play the major role in the recognition of the silverfish.

A relationship between chemical resemblance and aggression is well known in the nestmate recognition of ants. Workers of the Argentine ant *Linepithema humile *showed elevated aggression against conspecific workers that were chemically more distant, while conspecific workers with similar profiles were treated amicably [49]. Among myrmecophiles, the *Phengaris *(*Maculinea*) *alcon *caterpillar biosynthesises a "pre-adoption" profile and adoption of caterpillars happened faster with higher accuracy of chemical mimicry of the host [42]. The innate biosynthesis of CHCs by myrmecophiles means that the origins of mimetic CHCs and model CHCs are different, which allows coevolutionary arms races to shape the degree of mimicry as well as the discrimination abilities by ants [50]. As described above, the synthesis of particular key stimuli used to deceive the host may be selected for in these scenarios [50]. These colony-unspecific stimuli allow the caterpillars to be adopted by any colony of their respective hosts, accompanied by local adaptation on a population level. In contrast, acquisition through physical contact to the host, as demonstrated here, means that the mimetic compounds of the model and the mimic are of identical origin. Coevolutionary arms races operate differently in this case, selecting for mimics with effective ways of acquiring host CHCs (e.g., through rubbing behaviour in *M. ponerophila *or the consumption of host larvae in *Cosmophasis bitaeniata *[51]). In the host, selection favours defence mechanisms to prevent such "CHC pilfering" by parasitic myrmecophiles [52]. The present study indicates that such a coevolutionary arms race takes place between the host *L. distinguenda *and the myrmecophile *M. ponerophila*. Sufficient contacts between the silverfish and the host ants are required to refresh the mimetic compounds and to gain increased chemical resemblance to the host, in order to acquire the colony's current "gestalt odour", resulting in social acceptance.

Besides adaptive adjustments in mimetic CHCs, the presence of additional cuticular compounds that do not match the ants' current template could potentially be responsible for recognition of aliens in social insect societies, and such cues could also explain the observed attacks against isolated silverfish. Workers of the carpenter ant *Camponotus herculeanus*, for example, attacked nestmates if they possessed one additional, foreign compound on their cuticle [45]. However, in our experiments an acquisition of additional compounds during isolation treatments that could have been responsible for the observed aggression seems unlikely for several reasons. First, aggression towards isolated and non-isolated host workers was not different, indicating that their chemical profiles were not influenced by the treatment. Second, we did not detect any specific compounds in silverfish (neither among isolated nor among non-isolated individuals) that could be responsible for the aggression, but we cannot exclude effects of compounds that were undetectable by the GC-MS analysis that was used. However, the silverfish isolation control experiment (additional file 6) finally shows that isolated silverfish did not acquire additional compounds during isolation that elicit aggression. Individuals that were first isolated and then were given the chance to re-acquire host CHCs were attacked significantly less than silverfish that were only isolated, indicating that only mimetic host compounds were behaviourally active.

### Behavioural and morphological adaptations

Considering the level of integration a myrmecophile can achieve, we want to emphasise that mechanisms other than chemical integration may also play important roles, such as acoustic mimicry or behavioural and morphological adaptations [20,21,53]. The myrmecophilous cricket *Myrmecophilous formosanus*, for example, avoids ant attacks by swift movements [53]. *Malayatelura ponerophila *was also regularly observed escaping by quick movements (behavioural category "avoid" in additional file 4). About 75% of the isolated silverfish survived the observation period despite frequent ant attacks during escape. The limuloid (drop-shaped), scaled body of silverfish, with short appendages (antennae, cerci and praecerci) and retractable head, may also facilitate escaping ant attacks. The convergent evolution of the limuloid body form in unrelated myrmecophilous taxa provides strong evidence of its adaptive value [54]. These traits may also help *M. ponerophila *to survive ant attacks in natural nests and perhaps offer the possibility of invading new host colonies, albeit this is presumably a risky manoeuvre. *Malayatelura ponerophila *usually prefers central regions within natural nests where callows, pupae and larvae are located [26]. When individuals are able to reach this inner part of the nest, they are in a fairly safe place, which not only offers shelter and food, but also offers the possibility to steal the host's chemical profile by rubbing their surface on defenceless callows (see additional file 8: Video of *M. ponerophila*).

## Conclusion

In summary, we show that ant parasites can acquire CHCs directly from their host. Although elaborate behavioural adaptations may be required, the direct acquisition of host CHCs appears to be an evolutionarily parsimonious mechanism for taxonomically distant parasites such as *M. ponerophila*. Furthermore, our study reveals that the accuracy of chemical mimicry can be crucial for parasites of social insects to gain social acceptance. For *M. ponerophila*, regular replenishment of mimetic compounds increases survival because individuals with low chemical host resemblance are recognised and attacked frequently, sometimes captured and killed. Notably, the less frequently a silverfish replenishes its chemical profile (e.g., by failure to locate defenceless callows), the more difficult it becomes to remain unrecognised and to seek contact with the host ants.

## Authors' contributions

CvB and VW designed the study, acquired the data, analysed and interpreted the data and drafted the manuscript. SS identified the chemical compounds and revised the manuscript. R H revised the manuscript. All authors approved the final manuscript.

## Supplementary Material

Additional file 1 **Calculation of animal surface areas**. Calculation of surface areas of the bodies of silverfish, workers and isopods. Click here for file

Additional file 2 **Table of compounds**. Concentrations of 32 compounds that constituted 99.06% of the chemical profiles of workers (*N *= 44) across colonies evaluated by a similarity percentage analysis (SIMPER) on Bray-Curtis distances. In addition, concentrations of non-isolated (Sf 0 d; *N *= 63), six days isolated (Sf 6 d; *N *= 12) and nine day isolated silverfish (Sf 9 d; *N *= 56) across colonies are shown. Click here for file

Additional file 3 **Concentration of CHCs**. Shown is the total quantity of surface chemicals per area of non-isolated and isolated silverfish. Click here for file

Additional file 4 **Behavioural interactions in the social acceptance experiment**. Detailed information on behavioural interactions between silverfish and host ants across all colonies. Click here for file

Additional file 5 **NMDS plot of behavioural interactions between isolated and non-isolated silverfish and their host ants for colony 7**. Click here for file

Additional file 6 **Silverfish isolation control experiment**. Control for isolation treatment. Chemical similarities of silverfish to host workers and aggression toward the same individuals in colony 8. Click here for file

Additional file 7 **Worker control experiment**. Concentration of CHCs on non-isolated and isolated workers. Click here for file

Additional file 8 **Video of *M. ponerophila***. The video shows *M. ponerophila *together with host workers and brood in an artificial nest site. One silverfish individual (in the foreground) rubs its own body intensely on that of an ant worker, presumably to acquire host CHCs. Click here for file
