# Supplementary material for: Acquisition of chemical recognition cues facilitates integration into ant societies
Source: BMC Ecol. 2011 Dec 1;11:30. doi: 10.1186/1472-6785-11-30 (PMC3271039; doi:10.1186/1472-6785-11-30)
Supplement: Additional file 1 — Calculation of animal surface areas. Calculation of surface areas of the bodies of silverfish, workers and isopods. [file 1472-6785-11-30-S1.PDF]

Additional file 1 − Calculation of animal surface areas.

Silverfish. To estimate the surface area of a silverfish, we approximated their body form by dividing the body into different geometrical parts. The head area was estimated by calculating a quarter of the surface area of a sphere (dorsal surface) plus half the surface area of a cirlce (flat, ventral surface). The rest of the body was estimated by calculating half of the surface area of a cone (dorsal surface) plus the area of an isosceles triangle (flat, ventral surface). The picture of *M. ponerophila* shows that the simplified body form approximately matches the actual body form. Accordingly, the surface area of each silverfish was calculated using the following formula (Dorn et al. 2005):

$$\text{Surface area}_{\text{silverfish}} = \frac{4\pi r^2}{4} + \frac{\pi r^2}{2} + \frac{\pi r s}{2} + \frac{2hr}{2}$$

Overall, we measured the surface area of 180 individuals (including the 90 individuals used for calculating the surface concentration of CHCs). The median surface area was 13.48 mm^2 with a maximum of 19.71 mm^2 and a minimum of 2.44 mm^2. The data set shows no normal distribution (Shapiro-Wilk, $W = 0.98$, $P < 0.005$).

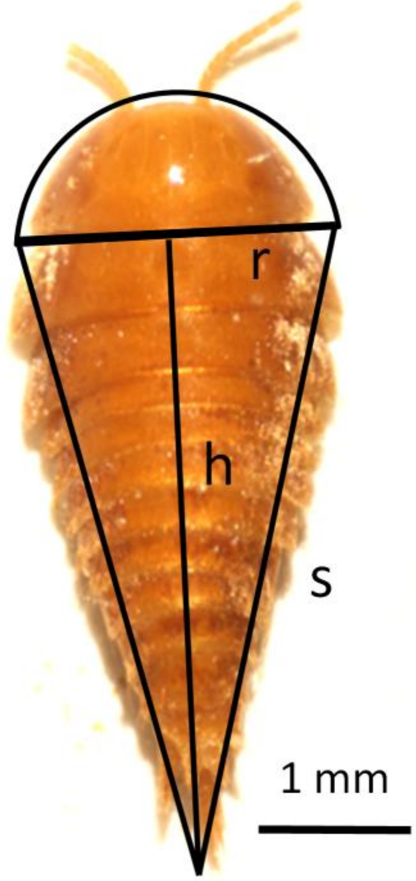

Ant workers. The worker's body shape was divided into geometrical parts to estimate their surface area. The surface area of the head, the allitrunk (thorax), the petiole and the gaster were calculated by using the approximation of a three-axis ellipsoid according to Thomson (http://de.wikipedia.org/wiki/Ellipsoid):

$$\text{Surface area}_{\text{ellipsoid}} = 4\pi\left[\frac{(ab)^{1.6}+(ac)^{1.6}(bc)^{1.6}}{3}\right]^{0.625}$$

The variables a, b and c correspond to the length, the breadth and the height of the respective ant body part. The lateral surface area of four circular cylinders was calculated to estimate the surface area of the legs consisting of the coxa, the femur, the tibia and the tarsus (the surface area of the trochanter was neglected).

$$\text{Surface area}_{\text{lateral area of cylinder}} = 2\pi rh = \pi dh$$

The variables d and h correspond to the length and breadth of the respective part of the ants' leg. We measured the surface area of 10 individuals. The median surface area was 78.24 mm^2 with a maximum of 83.09 mm^2 and a minimum of 71.76 mm^2. The data set shows a normal distribution (Shapiro-Wilk, $W = 0.92$, $P = 0.400$).

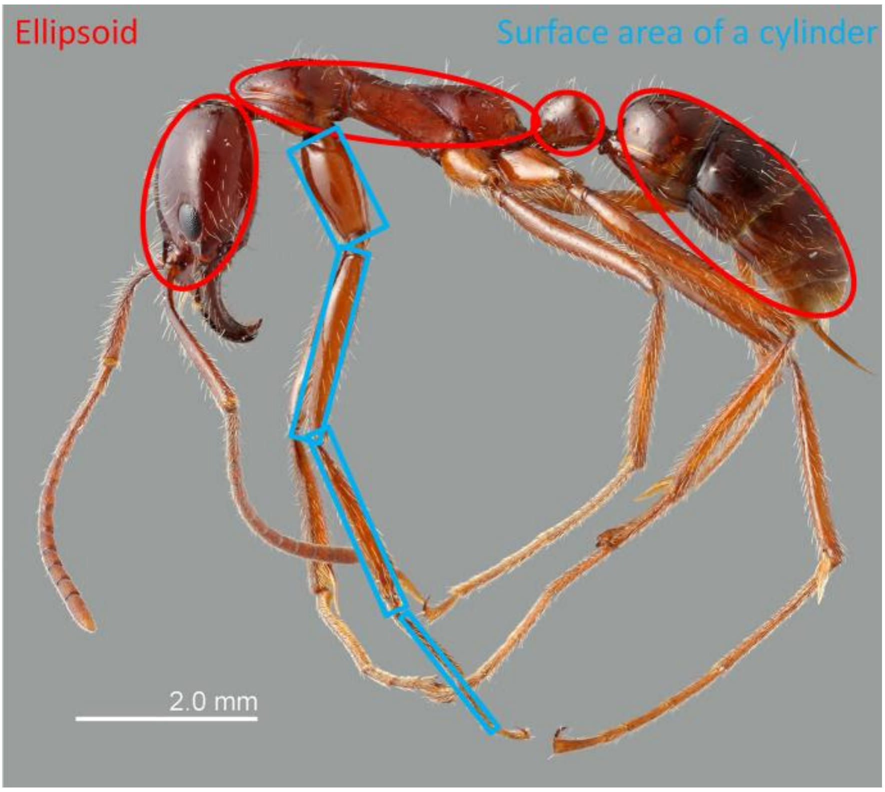

© Munetoshi Maruyama

Isopods. The area of an isopod's dorsal surface was calculated by halving the surface area of a three-axis ellipsoid according to Thomson (see above). The area of the isopod's ventral surface was determined by applying the formula of an ellipse:

$\text{Surface area}_{\text{ellipse}} = 2\pi ab$

The variables a and b represent half of the ellipse's major and minor axes, respectively.

The median surface area of isopods ($N = 22$) was 42.69 mm^2 with a maximum of 95.86 mm^2 and a minimum of 27.04 mm^2. The data set shows no normal distribution (Shapiro-Wilk, $W = 0.83$, $P = 0.017$).

Reference:

Dorn H-J, Freudigmann H, Herbst M: *Formelsammlung Mathematik. Gymnasium: Sekundarstufe I und II.*, Ernst Klett Verlag, Stuttgart; 2005.
