# Supplementary material for: Acquisition of chemical recognition cues facilitates integration into ant societies
Source: BMC Ecol. 2011 Dec 1;11:30. doi: 10.1186/1472-6785-11-30 (PMC3271039; doi:10.1186/1472-6785-11-30)
Supplement: Additional file 2 — Table of compounds. Concentrations of 32 compounds that constituted 99.06% of the chemical profiles of workers (N = 44) across colonies evaluated by a similarity percentage analysis (SIMPER) on Bray-Curtis distances. In addition, concentrations of non-isolated (Sf 0 d; N = 63), six days isolated (Sf 6 d; N = 12) and nine day isolated silverfish (Sf 9 d; N = 56) across colonies are shown. [file 1472-6785-11-30-S2.PDF]

Additional file 2 – Table of compounds

Concentrations of 32 compounds that constituted 99.06% of the chemical profiles of workers (N = 44) across colonies evaluated by a similarity percentage analysis (SIMPER) on Bray-Curtis distances. In addition, concentrations of non-isolated (Sf 0 d; N = 63), six days isolated (Sf 6 d; N = 12) and nine days isolated silverfish (Sf 9 d; N = 56) across colonies are shown. Abbreviation: SE = standard error

	Concentrations [µg/mm^2 ± SE]			
**Compound**	**Workers**	**Sf 0 d**	**Sf 6 d**	**Sf 9 d**
Nonacosene (A)	18.685 ± 1.119	7.254 ± 0.926	3.222 ± 0.782	4.715 ± 0.780
Tricosane	17.830 ± 0.862	14.229 ± 1.000	4.441 ± 0.895	5.043 ± 0.655
Heptacosene (A)	13.618 ± 1.039	5.318 ± 0.702	1.373 ± 0.400	3.558 ± 0.618
Pentacosene (A)	11.812 ± 1.025	3.522 ± 0.367	0.536 ± 0.127	1.808 ± 0.294
Pentacosadiene	8.280 ± 0.489	3.159 ± 0.551	0.843 ± 0.187	1.583 ± 0.342
Hentriacontene	6.016 ± 0.620	0.502 ± 0.084	0.076 ± 0.045	0.078 ± 0.026
Pentacosene (B)	5.686 ± 0.381	2.321 ± 0.305	0.770 ± 0.167	1.289 ± 0.218
Heptacosadien	5.155 ± 0.496	1.135 ± 0.269	0.092 ± 0.062	0.482 ± 0.203
Heptacosene (B)	3.683 ± 0.554	0.810 ± 0.113	0.207 ± 0.070	0.485 ± 0.090
Pentacosane	3.512 ± 0.192	2.658 ± 0.235	0.923 ± 0.191	0.977 ± 0.142
Nonacosene (B)	1.958 ± 0.206	0.170 ± 0.039	0.085 ± 0.052	0.028 ± 0.017
Pentacosene (C)	1.383 ± 0.111	0.774 ± 0.144	0.163 ± 0.084	0.316 ± 0.077
Tricosene (C)	1.072 ± 0.175	0.826 ± 0.162	0.118 ± 0.061	0.230 ± 0.059
11-Methylpentacosane	1.036 ± 0.091	0.206 ± 0.034	0.113 ± 0.055	0.052 ± 0.017
Decyloctanoate	0.904 ± 0.100	0.004 ± 0.003	-	0.007 ± 0.007

Tritriacontene	0.847 ± 0.125	0.001 ± 0.001	-	-
Tricosene (B)	0.833 ± 0.074	0.093 ± 0.023	0.008 ± 0.008	0.002 ± 0.002
Nonacosadiene	0.832 ± 0.159	-	0.025 ± 0.025	-
11-Methylheptacosane	0.806 ± 0.063	0.239 ± 0.035	0.309 ± 0.131	0.062 ± 0.018
Tetracosane	0.651 ± 0.037	0.503 ± 0.074	0.156 ± 0.047	0.149 ± 0.032
Decyl decanoate	0.610 ± 0.072	0.001 ± 0.001	-	0.002 ± 0.011
Octacosene (A)	0.505 ± 0.064	-	-	-
Multiple methylated hentriacontenes	0.494 ± 0.102	-	-	-
13- and 15- Methylhentriacontanes	0.450 ± 0.087	-	-	-
6,9,12,15-Heptacosatetraene	0.432 ± 0.049	0.033 ± 0.012	-	0.005 ± 0.003
13- and 15- Methylnonacosanes	0.394 ± 0.056	0.001 ± 0.001	0.056 ± 0.052	-
Docosane	0.307 ± 0.018	0.449 ± 0.136	0.042 ± 0.013	0.005 ± 0.005
Octacosene (B)	0.279 ± 0.058	0.003 ± 0.003	-	-
Hexacosene (B)	0.254 ± 0.057	0.001 ± 0.000	-	-
Heneicosane	0.196 ± 0.021	0.280 ± 0.071	0.023 ± 0.008	0.001 ± 0.000
9-Methyltricosane	0.132 ± 0.010	0.001 ± 0.001	-	-
