# Supplementary material for: Acquisition of chemical recognition cues facilitates integration into ant societies
Source: BMC Ecol. 2011 Dec 1;11:30. doi: 10.1186/1472-6785-11-30 (PMC3271039; doi:10.1186/1472-6785-11-30)

Additional file 3 – Concentration of CHCs.

The graph shows the total quantity of surface chemicals per area of non-isolated and isolated silverfish. One outlier of a non-isolated silverfish in colony 1 is not shown in the graph for better visualisation (outlier = 171 ng/mm2). Significant differences between groups were evaluated by PERMANOVA (****P* < 0.001; ***P* < 0.010; n.s. = not significant). Median (+= mean), quartiles (boxes), 10th and 90th percentiles (whiskers), and outliers (♦ = outlier) are shown. Abbreviations: No iso = no isolation, d iso = days isolated

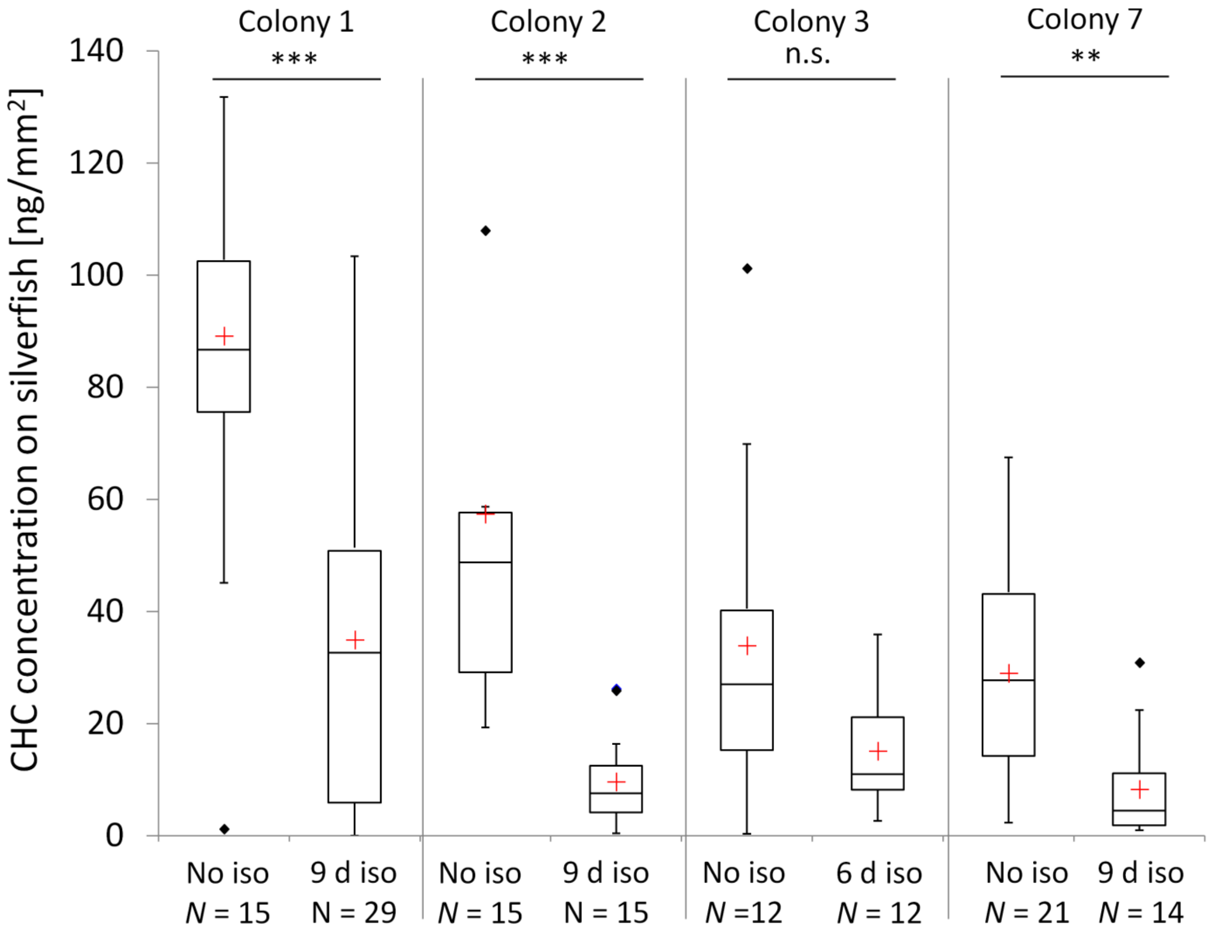

Supplement: Additional file 3 — Concentration of CHCs. Shown is the total quantity of surface chemicals per area of non-isolated and isolated silverfish. [file 1472-6785-11-30-S3.PDF]
