# Supplementary material for: Acquisition of chemical recognition cues facilitates integration into ant societies
Source: BMC Ecol. 2011 Dec 1;11:30. doi: 10.1186/1472-6785-11-30 (PMC3271039; doi:10.1186/1472-6785-11-30)
Supplement: Additional file 4 — Behavioural interactions in the social acceptance experiment. Detailed information on behavioural interactions between silverfish and host ants across all colonies. [file 1472-6785-11-30-S4.PDF]

Additional file 4 – Behavioural interactions in the social acceptance experiment.

**Interaction**	**Colony 4** No isolation $N = 7$		**Colony 4** 6d isolation $N = 6$		**Colony 5** No isolation $N = 18$		**Colony 5** 6d isolation $N = 22$		**Colony 7** No isolation $N = 21$		**Colony 7** 9d isolation $N = 12$	
Ignored	59<br>9	A	50<br>8.5	A	219<br>12.5	A	101<br>4	B	267<br>10	A	82<br>6	B
Groomed	6<br>0	A	0<br>0	A	8<br>0	A	1<br>0	A	0<br>0	A	1<br>0	A
Avoid	4<br>0	A	28<br>5.5	B	35<br>0	A	170<br>2	B	98<br>4	A	149<br>12.5	B
Antennated	3<br>0	A	15<br>2	B	17<br>0	A	45<br>1	B	34<br>1	A	33<br>3	B
Unnoticed	260<br>40	A	156<br>25	B	623<br>35	A	326<br>14	B	659<br>31	A	257<br>21	B
Chased	1<br>0	A	30<br>4.5	B	1<br>0	A	185<br>8	B	19<br>1	A	34<br>3	B
Snapped	0<br>0	A	23<br>3.5	B	6<br>0	A	291<br>11.5	B	25<br>1	A	39<br>3	B
Stung	0<br>0	A	6<br>1	B	1<br>0	A	24<br>1	B	0<br>0	A	1<br>0	A

The upper number in each array represents the sum and the lower number indicates the median of the corresponding interaction. Different capital letters depict significant differences ($P < 0.05$) for a given behavioural interaction evaluated by PERMANOVA. We did not apply statistics for colony 6 because none of the isolated individuals completed the standardized number of 50 ant contacts (Tab. 1). Abbreviations: N = number of silverfish, 6d = six days, 9d = nine days
