# Supplementary material for: Acquisition of chemical recognition cues facilitates integration into ant societies
Source: BMC Ecol. 2011 Dec 1;11:30. doi: 10.1186/1472-6785-11-30 (PMC3271039; doi:10.1186/1472-6785-11-30)

Additional file 5 − NMDS plot of behavioural interactions between isolated and non-isolated silverfish and their host ants for colony 7.

Each data point represents 50 encounters of a silverfish individual with a host worker. Arrows represent the relative contributions of behaviours (see Table 2) to data separation, whereby the length indicates the importance (observed frequency). For clarity, the origin of arrows is not centred in the plot. “Stress” is a quality measure of NMDS.

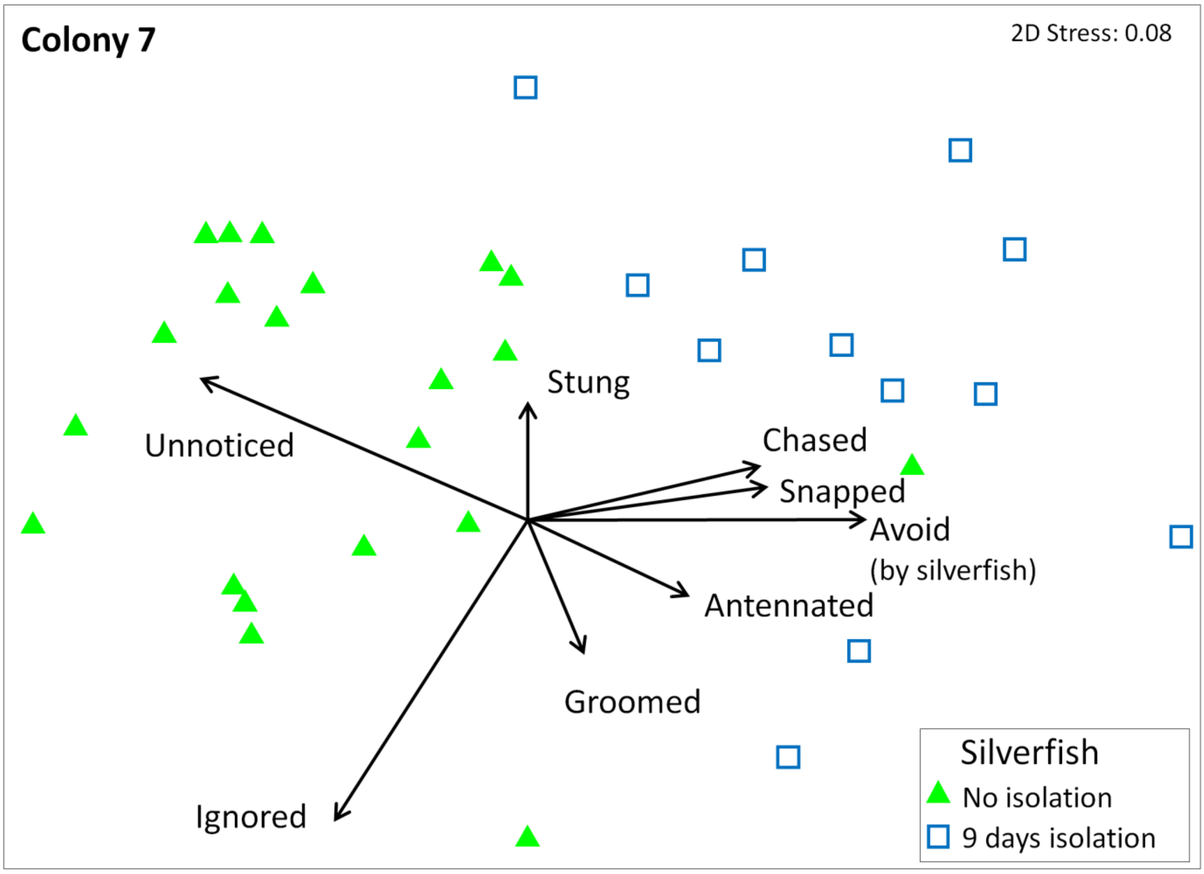

Supplement: Additional file 5 — NMDS plot of behavioural interactions between isolated and non-isolated silverfish and their host ants for colony 7. [file 1472-6785-11-30-S5.PDF]
