# Supplementary material for: Acquisition of chemical recognition cues facilitates integration into ant societies
Source: BMC Ecol. 2011 Dec 1;11:30. doi: 10.1186/1472-6785-11-30 (PMC3271039; doi:10.1186/1472-6785-11-30)

Additional file 6 – Silverfish isolation control experiment.

Chemical similarities of silverfish to host workers and aggression toward the same individuals in colony 8. Differences between groups were evaluated by PERMANOVA (****P* < 0.001). Median (+ = mean), quartiles (boxes), 10th and 90th percentiles (whiskers), and outliers (♦ = outlier) are shown. Abbreviations: 6 d iso = 6 days isolation, 1 d Wo = silverfish kept one day together with host workers

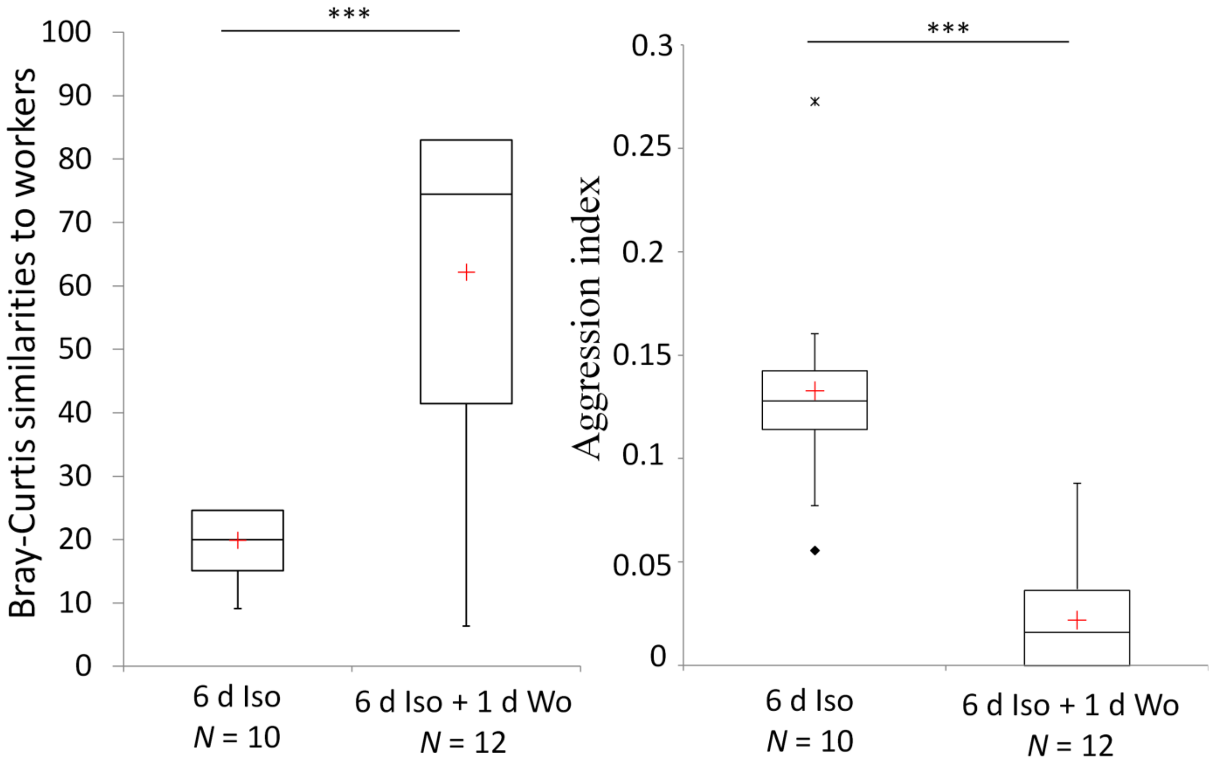

Supplement: Additional file 6 — Silverfish isolation control experiment. Control for isolation treatment. Chemical similarities of silverfish to host workers and aggression toward the same individuals in colony 8. [file 1472-6785-11-30-S6.PDF]
