# Supplementary material for: Acquisition of chemical recognition cues facilitates integration into ant societies
Source: BMC Ecol. 2011 Dec 1;11:30. doi: 10.1186/1472-6785-11-30 (PMC3271039; doi:10.1186/1472-6785-11-30)

Additional file 7 − Worker control experiment.

Concentration of CHCs on non-isolated and isolated workers. Significant differences between groups were evaluated by PERMANOVA (**P* < 0.05; ****P* < 0.001; n.s. = not significant). Median (+ = mean), quartiles (boxes), 10th and 90th percentiles (whiskers), and outliers (♦ = outlier) are shown. Abbreviations: 9 d iso = nine days isolation

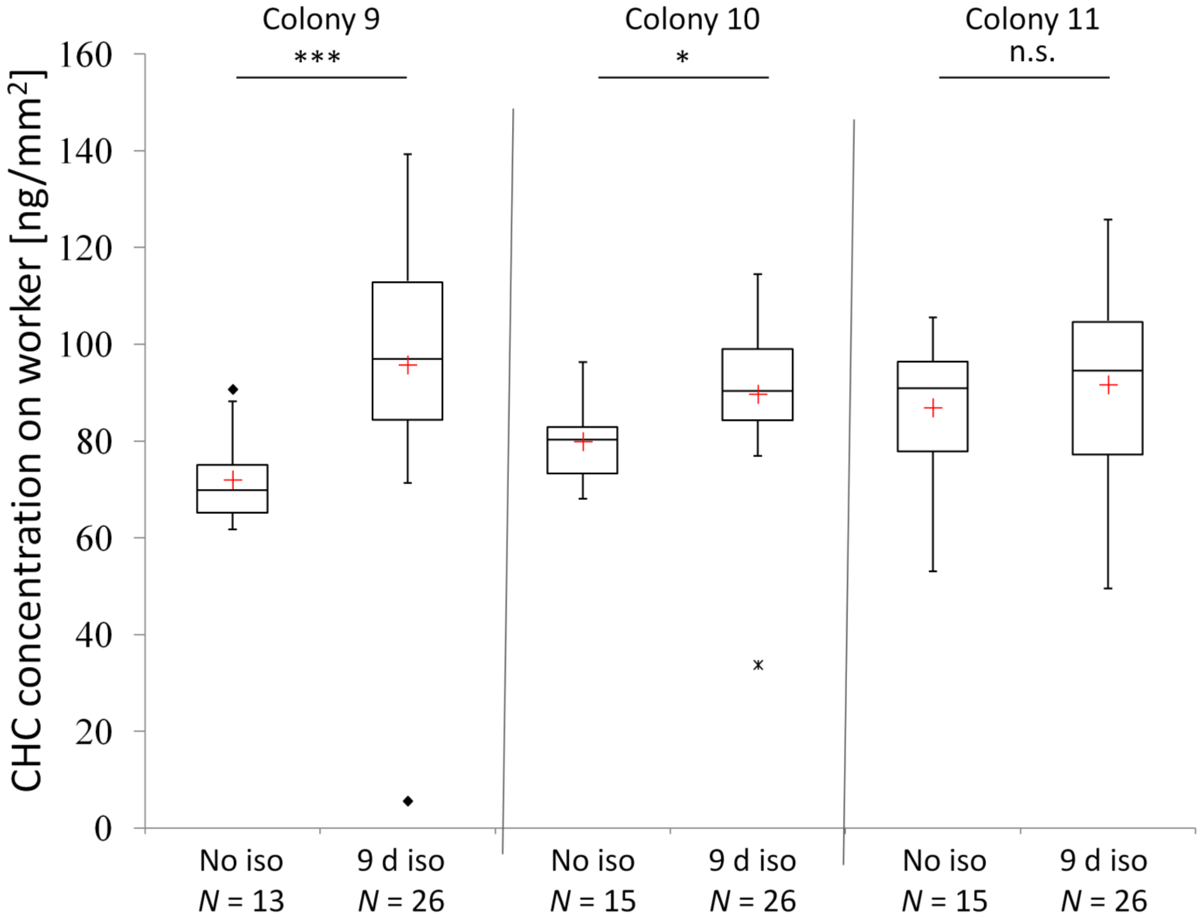

Supplement: Additional file 7 — Worker control experiment. Concentration of CHCs on non-isolated and isolated workers. [file 1472-6785-11-30-S7.PDF]
